# First reported case of a collision tumor composed of pancreatic adenocarcinoma and retroperitoneal liposarcoma: a case report

**DOI:** 10.1186/s12885-018-5151-6

**Published:** 2018-12-12

**Authors:** Denise Buchner, Lena Hieggelke, Heike Löser, Christiane Josephine Bruns, Alexander Quaas, Markus Philipp Hussein Ghadimi

**Affiliations:** 1 Department of General, Visceral and Tumor Surgery, University Hospital of Cologne, Kerpener Strasse 62, 50937 Cologne, Germany; 2 University Hospital of Cologne, Institute of Pathology, Cologne, Germany

**Keywords:** Collision tumor, Pancreatic adenocarcinoma, Liposarcoma, MDM2 amplification

## Abstract

**Background:**

Collision tumors are rare cases with two different tumor entities growing synchronously. While adenocarcinoma of the pancreas is the most common pancreatic tumor with an incidence of 10 per 100.000, retroperitoneal liposarcoma remains very rare. This is the first report of a collision tumor between these two tumor entities.

**Case presentation:**

Demographic details:

The tumor was diagnosed in a 64 male Caucasian patient. Besides atrial fibrillation, arterial hypertension and a hypothyroidism there is no relevant medical history especially no history of cancer.

Clinical details:

During a routine check-up an unclassified tumor of the pancreatic tail was diagnosed. The lab showed no pathologies. Tumor markers were negative for carbohydrate antigen 19–9 and 72–4 (CA 19–9, CA 72–4) and carcinoembryonic antigen (CEA). Alpha-fetoprotein (AFP) and neuron specific enolase (NSE) were both elevated (AFP 97kU/l, (< 5,8kU/l) and NSE 30,0 μg/l (16,4 μg/l)). A computed tomography-guided core needle biopsy was performed which revealed a low-grade liposarcoma (G1). A CT scan showed no metastases. A surgical resection was recommended by the interdisciplinary tumor board.

Interventions: A systematic left sided retroperitoneal compartment resection including en-bloc-left sided pancreatectomy, splenectomy, nephrectomy, hemicolectomy, adrenalectomy, partial gastrectomy and partial resection of the diaphragm was performed. Pathology revealed a collision tumor consisting of pancreatic adenocarcinoma that was classified pT3, pN2 (11/33 ece+) L1 V0 Pn0, R0; G2 [UICC Stage III] and a liposarcoma pT2, pN0 (0/33) L0 V0 Pn0, G1 [UICC Stage Ib].

The postoperative tumor board recommended an adjuvant chemotherapy with gemcitabine and capecitabine for the locally advanced pancreatic adenocarcinoma.

Outcome: At the latest follow-up (1 year after surgery) the patient was in good clinical condition and without evidence of tumor recurrence.

**Conclusion:**

Collision tumors are rare and difficult to diagnose. This is the first description of a collision tumor composed of pancreatic adenocarcinoma and retroperitoneal liposarcoma.

The reported case demonstrates that inconsistent diagnostic results (e.g. imaging and pathology) should raise suspicion concerning the diagnosis. Awareness of these rare cases might protect us from underdiagnosing patients and therefore leading to better patient care.

There is evolving evidence that will lead to more personalized treatment options for somatic BRCA mutated pancreatic cancer.

**Electronic supplementary material:**

The online version of this article (10.1186/s12885-018-5151-6) contains supplementary material, which is available to authorized users.

## Background

Collision tumors are rare cases with two tumor entities raised from two different cell types occurring synchronously in the same organ or in close anatomic location. While adenocarcinoma of the pancreas is the most common pancreatic tumor with an incidence of 10 per 100.000, retroperitoneal liposarcoma remains very rare. This is the first report of a collision tumor composed of these two tumor entities.

## Case presentation

### Demographic details

The tumor was diagnosed in a 64 year old, male Caucasian patient during a routine check-up.

Medical history: Besides atrial fibrillation, arterial hypertension and a hypothyroidism there is no relevant medical history especially no history of cancer.

### Symptoms and signs

**Fig. 1:**
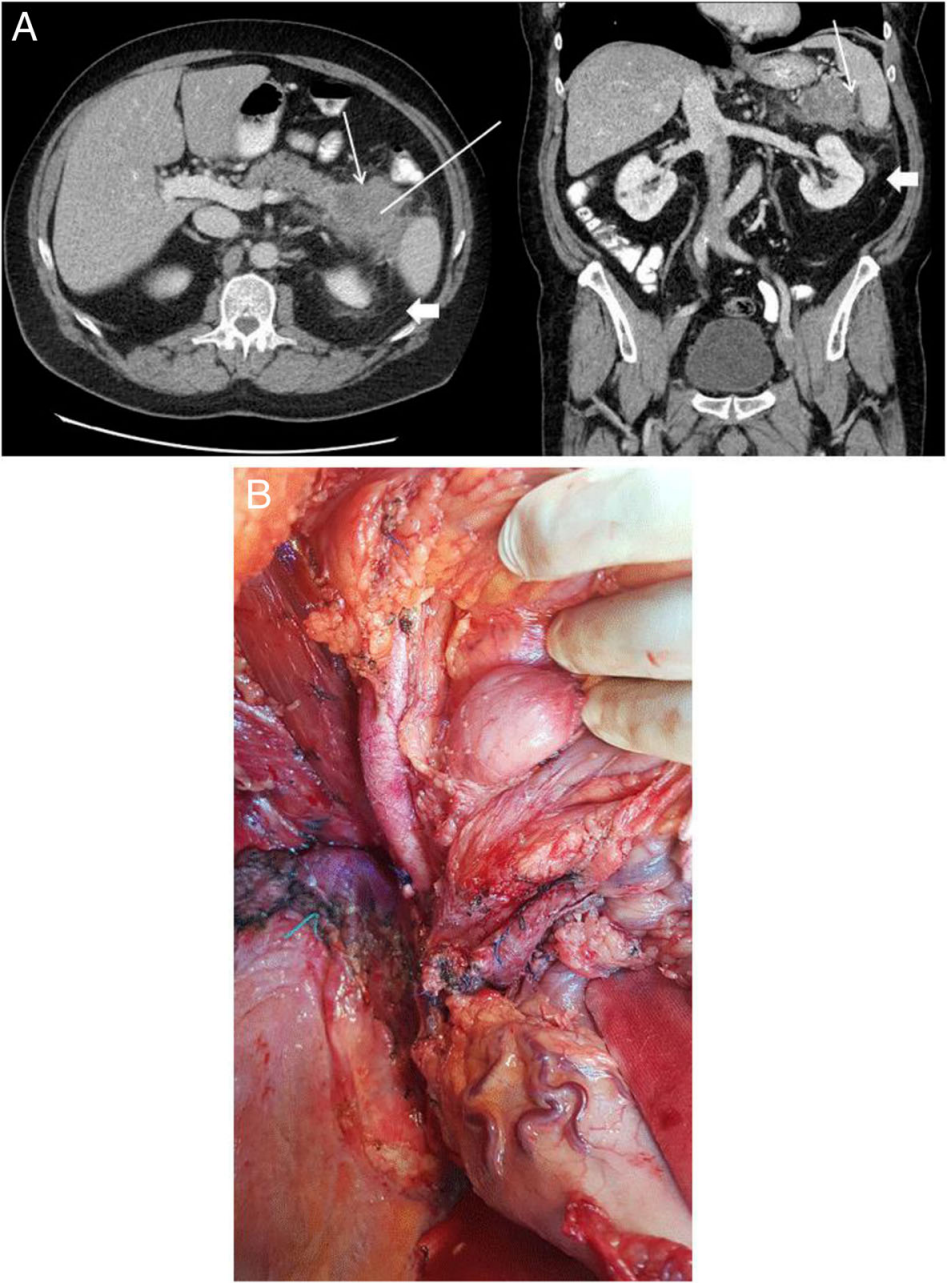
**a** preoperative CT-scan (left – axial, right – coronal), showing a central hypodense tumor mass of 6,4 × 6,8 cm in the pancreatic tail (marked with white arrow) and a perifocal, diffuse fibrotic enhancement of the retroperitoneal fat (marked with white arrow head). The corridor of the CT core needle biopsy is marked with a white line; **b** intraoperative situs showing

During a routine check-up the patients physician performed a sonography when he detected an unclassified tumor of the pancreatic tail (see also Additional file 1). The lab showed no pathologies. Tumor markers were negative for CA 19–9, CA 72–4 and CEA. Alpha-fetoprotein and NSE were both elevated (AFP 97kU/l, (< 5,8kU/l) and NSE 30.0 μg/l (16.4 μg/l)). A CT scan showed no metastases (Fig. 1a). A core needle biopsy was performed to define the tumor entity. The histology of the core needle biopsy extracted from the pancreatic tail area showed fibrotic fat with atypical adipocytes. The fluorescence-in-situ-hybridization (FISH) revealed MDM2-cluster amplification. Thus the diagnosis of a de-differentiated liposarcoma (DDL) was made.

With this histological diagnosis the solid tumor at the pancreatic tail was considered to be a dedifferentiated part of the liposarcoma. A primary surgical resection was recommended by the interdisciplinary tumor board.

### Treatment

A systematic left sided retroperitoneal compartment resection including en-bloc-left sided pancreatectomy, splenectomy, nephrectomy, hemicolectomy, adrenalectomy, partial gastrectomy and partial resection of the diaphragm were performed (Fig. 1b).

### Outcome

Having recovered from surgery and an anastomosis leak of the colon, the patient is now well. So far there were no signs of tumor recurrence.

### Pathology

The resected multivisceral specimen measured 35 × 19 × 12 cm and weighed 2520 g. The macroscopic examination showed a firm, inhomogeneous, yellowish gray, 7.6 cm diameter tumor between the pancreatic tail and spleen. The further investigation of the bordering retroperitoneal fat tissue revealed a 5.6 cm large area slightly solidified, marginally lobulated, yellow, lipomatous tumor without discrete margins.

**Fig. 2:**
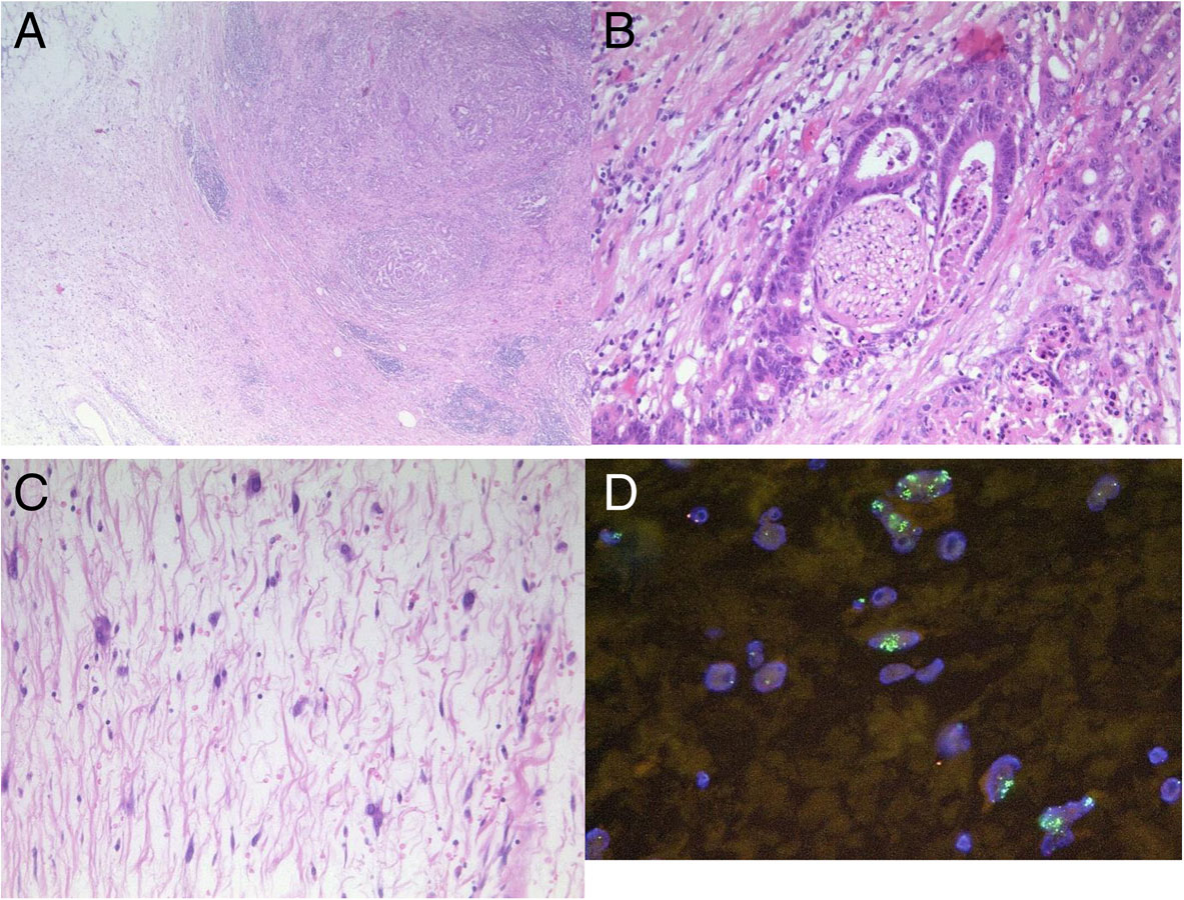
**a** Overview of the pancreatic adenocarcinoma colliding with the liposarcoma (Hematoxylin-Eosin (HE), original magnification × 50); **b** Perineural invasion of the pancreatic adenocarcinoma (HE, original magnification × 100); **c** Liposarcoma in detail (HE, original magnification × 200); **d** FISH mdm2 cluster amplification (green signals)

Microscopic examination (Figs. 2a-d)  of the tumor mass in the pancreatic tail showed an epithelial tumor with glandular structure, associated with a desmoplastic stroma reaction, continuously infiltrating the fat tissue and lymph nodes. The tumor cells with increased mitotic activity, nuclear pleomorphism and prominent cell nucleoli showed a cribriform and haphazard growth pattern. Furthermore there were necrotic areas and perineural as well as lymphatic vessel invasion definable (Fig. 2b). A tumor infiltration in spleen, kidney or colon was not detectable. In the directly adjacent retroperitoneum atypical lipomatous mesenchymal cells and multinucleated as well as vacuolated lipoblasts were found (Fig. 2c). These cells had been known from the punch biopsy and were also MDM2-cluster amplified, representing a liposarcoma of the retroperitoneum (Fig. 2d). Parts of this tumor approximated < 0.1 cm to the surgical margins. Taken together there was a completely resected pancreatic ductal adenocarcinoma and a well-differentiated liposarcoma with extended growth to the peritoneal margins. Additional molecular pathological investigation revealed a pathogenic category 5 mutation of pathogenic BRCA 2 of the adenocarcinoma. The pancreatic adenocarcinoma was classified as pT3, pN2 (11/33 ece+) L1 V0 Pn0, R0; G2 [UICC Stage III] with a drugable BRCA mutation and the liposarcoma as pT2, pN0 (0/33) L0 V0 Pn0, G1 [UICC Stage Ib]. Further treatment: The interdisciplinary tumor board recommended an adjuvant chemotherapy with the standard of treatment, being Gemcitabine and Capecitabine. The patient recovered well after surgery and is currently cancer free under frequent follow up for the last year. The latest follow-up examination revealed no evidence of tumor recurrence.

## Discussion

This is the first description of a collision tumor composed of pancreatic adenocarcinoma and a retroperitoneal liposarcoma. A PubMed search of “collision tumor” showed by the time 1054 publications. Most of the tumors are described in neurosurgery, dermatology and urology. Looking at visceral surgery most collision tumors are described at the stomach (e.g. GIST and adenocarcinoma). There are only 11 publications describing collision tumors with pancreatic involvement with a collision between ductal adenocarcinoma and neuroendocrine tumor both of the pancreas being the most frequently described [1–11]. There are only 8 publications describing a collision tumor with any sarcoma involved [10–17]. In those cases liposarcoma and leiomyosarcoma are the two most frequently mentioned entities.

In our case, pre-interventional CT showed a solid hypodense mass indicating a pancreatic neoplasm (Fig. 1a). We first diagnosed the liposarcoma by core needle biopsy. The liposarcoma belongs to soft tissue sarcomas (STS) and is the most common type of STS in the retroperitoneum (63%) [18]. While well differentiated liposarcoma (DL) is a locally aggressive neoplasia almost incapable of systemic spread, De-differentiated liposarcoma (DDL) metastasizes in 10–15% of cases. All types of WDL share the same genetic aberration. Amplification of the chromosomal region 12q13–15 containing the proto-oncogenes MDM2, CDK4 and HMGA2 is a pathognomonic feature. The circumstance that no pancreatic tissue could be found in the biopsy-specimen should therefore raise attention. The patient received a systematic left sided retroperitoneal compartment resection (Fig. 1b). The medial dissection plane was on the abdominal aorta, therefore sufficient for both, the WDL and the pancreatic ductal carcinoma. Yet, a systematic lymph node dissection as needed for the pancreatic carcinoma was not performed. In such cases the tumor should be treated as a pancreatic carcinoma with respect to the systematic lymph node dissection and the resection should be extended to the retroperitoneum as needed for retroperitoneal sarcomas. Otherwise, if the pancreatic adenocarcinoma was already diagnosed by biopsy, it would be conceivable that the resection had not been carried out to this extent. In this case, a liposarcoma would have been more difficult to detect.

In this special clinical context with a well differentiated liposarcoma and a nodal metastasized pancreatic adenocarcinoma the pancreatic cancer is crucial for the prognosis, so adjuvant treatment focusses on the adenocarcinoma.

Molecular-pathologic examination revealed a pathogenic BRCA 2 mutated pancreatic ductal adenocarcinoma (PDA). PDA remains one of the malignancies with the worst prognosis [19]. While most PDAs are thought to be sporadic, approximately 5 to 10% occur hereditarily. Multiple syndromes and diseases have been associated with an increased risk of developing pancreatic cancer, including hereditary breast and ovarian cancer with mutation of breast cancer 1 (*BRCA*1) and breast cancer 2 (BRCA2) [20], which lead to a deficiency in deoxyribonucleic acid (DNA) damage repair (DDR). Especially the homologous recombination repair (HRR) of DNA double strand is defective in BRCA deficiency, resulting in chromosomal instability and tumorigenesis [21]. Another family of DNA repair enzymes comprises Poly(ADP-ribose) polymerases (PARPs). PARPs are involved in crucial complementary repair processes. While HRR is ineffective in BRCA mutated cancer cells, PARP in turn plays a major role in repairing damaged DNA to maintain cell survival. A promising feature in the therapy of BRCA mutated cancers are PARP inhibitors and chemotherapeutic agents that induce DNA damage in the presence of impaired DNA repair [22].

According to german oncology guidelines, adjuvant treatment with Gemcitabine was recommended [23]. We plan an extension of the adjuvant chemotherapy with Capecitabine, according to the findings of the ESPAC-4-Study. Especially after negative margin resections, this study shows a survival benefit after treatment with Gemcitabine and Capecitabine compared to Gemcitabine monotherapy [24].

Because of the extended lymph node involvement of the pancreatic ductal adenocarcinoma the risk of recurrence was considered to be high [25, 26]. In case of tumor recurrence we plan a treatment with platin-based chemotherapy or specific PARP inhibitors. For BRCA2 mutated pancreatic cancer there is in vitro and in vivo data suggesting a higher sensitivity of tumor cells to DNA-crosslinking agents [27, 28]. Golan et al. showed in a retrospective study in patients with BRCA1/2 mutation a survival benefit for patients treated with platin based chemotherapy compared to non-platinum chemotherapy (22 vs. 9 months) [29].

PARP inhibitors have been proven to be a new treatment option for patient with germline-mutated ovarian and breast cancer [30, 31]. For ovarian cancer the European medicines Agency (EMA) has approved treatment of somatically mutated tumors with olaparib [32]. A phase II trial of olaparib monotherapy for patients with BRAC1/2 germline-mutated advanced pancreatic cancer showed 36,4% of patients to be progression-free at 6 months and 40,9% to be alive at 12 months [33]. At the moment there is one ongoing study in the USA which is investigating the benefit of a olaparib treatment for patients with a BRAC1/2 somatically-mutated pancreatic [34]. So far there is no evidence for the treatment with specific PARP inhibitors in the adjuvant setting [23].

There is no evidence for the use of adjuvant radiotherapy or chemotherapy after a R0-resection of a G1 liposarcoma, especially when as in our case the pancreatic ductal adenocarcinoma is the prognosis defining entity.

## Conclusion

Collision tumors are rare and difficult to diagnose. The reported case demonstrates that inconsistent diagnostic results (such as imaging and pathology) should raise suspicion concerning the diagnosis. Awareness of these rare cases might protect us from underdiagnosing patients and therefore leading to better patient care.

There is evolving evidence that will lead to more personalized treatment options for somatic BRCA mutated pancreatic cancer.

## Additional file

Additional file 1: The “timeline” visualizes the course of treatment of the patient from the time of first detection of the tumor by the patients general practioner to the time of last follow-up. (DOCX 42 kb)

## Abbreviations

CA 19–9: Carbohydrate antigen 19–9
CA 72–4: Carbohydrate antigen 72–4
CEA: Carcinoembryonic antigen
AFP: Alpha-fetoprotein
NSE: Neuron specific enolase
CT: Computed tomography
FISH: Fluorescence in-situ hybridization
MDM2: Mouse double minute 2 homolog
DDL: De-differentiated liposarcoma
BRCA 2: Breast cancer type 2 susceptibility protein
UICC: Union internationale contre le cancer
WDL: Well differentiated liposarcoma
STS: Soft tissue sarcoma
PLS: Pleomorphic liposarcoma
CDK 4: Cyclin-dependent kinase 4
HMGA2: High-mobility group AT-hook 2
PDA: Pancreatic ductal adenocarcinoma
DNA: Deoxyribonucleic acid
DDR: Deoxyribonucleic acid damage repair
HRR: Homologous recombination repair
PARP: Poly(ADP-ribose) polymerases
EMA: European medicines Agency
