# Supplementary material for: First reported case of a collision tumor composed of pancreatic adenocarcinoma and retroperitoneal liposarcoma: a case report
Source: BMC Cancer. 2018 Dec 12;18:1243. doi: 10.1186/s12885-018-5151-6 (PMC6292103; doi:10.1186/s12885-018-5151-6)
Supplement: Supplementary file 1 — The “timeline” visualizes the course of treatment of the patient from the time of first detection of the tumor by the patients general practioner to the time of last follow-up. (DOCX 42 kb) [file 12885_2018_5151_MOESM1_ESM.docx]

**Timeline**

**11 / 2017**

Start of adjuvant chemotherapy

**10 / 2017**

3^rd^ Tumorboard recommends adjuvant chemotherapy with gemcitabine and capecitabine

Histopathology of the surgical specimen reveals a collision tumor consisting of PDA and DDL

Operation: Systematic left-sided retroperitoneal compartment resection

**09 / 2017**

Biopsy reveals a De-differentiated liposarcoma

2^nd^ Tumorboard recommends primary resection

1. Computed tomography
2. Tumor Board presentation, recommendation to obtain histopathology by core-needle biopsy.
3. CT-guided Biopsy

**08 / 2017**

Referral and Initial presentation in outpatient clinic of the university hospital cologne

**07 / 2017**

**07 / 2017**

**Diagnosis** of a left-sided retroperitoneal mass at the pancreatic tail during a routine ultrasound check-up at his general practitioner (GP)

CT-scan

Last Follow-up without evidence of recurrent disease

**03 / 2018**
